# Polycation stabilization of graphene suspensions

**DOI:** 10.1186/1556-276X-6-493

**Published:** 2011-08-16

**Authors:** Kamran ul Hasan, Mats O Sandberg, Omer Nur, Magnus Willander

**Affiliations:** 1 Department of Science and Technology (ITN), Linköping University, Campus Norrköping, SE-601 74 Norrköping, Sweden; 2 Acreo AB Bredgatan 34, SE-602 21 Norrköping, Sweden

## Abstract

Graphene is a leading contender for the next-generation electronic devices. We report a method to produce graphene membranes in the solution phase using polymeric imidazolium salts as a transferring medium. Graphene membranes were reduced from graphene oxides by hydrazine in the presence of the polyelectrolyte which is found to be a stable and homogeneous dispersion for the resulting graphene in the aqueous solution. A simple device with gold contacts on both sides was fabricated in order to observe the electronic properties.

## Introduction

The unique physical, electronic, and optical properties of graphene have been reported many times [1-4] and promise a wide variety of applications. Different methods have been adopted for obtaining graphene, e.g., mechanical exfoliation of graphite [5], epitaxial growth [6], and chemical exfoliation in different solutions [3,7-9]. A very promising route for the bulk production of the graphene sheets can be chemical reduction and dispersion of graphene in aqueous solutions.

Two steps are involved in making water dispersible graphene: (1) first chemical oxidation of graphite to hydrophilic graphite oxide and (2) exfoliating it into graphene oxide (GO) sheets in aqueous solution. GO sheets are graphene sheets having oxygen functional groups. These GO sheets are prevented from agglomeration by electrostatic repulsion alone [10]. The insulating GO can easily be reduced to highly conducting graphene by hydrazine reduction. However, the reduction of GO soon leads to agglomeration, while a stable dispersion is key to the possibility of large-scale processing. Polymeric imidazolium salts can be a good way to form a stable dispersion of graphene.

Organic salts based on the imidazolium moiety are an interesting class of ions. Low molecular weight imidazolium salts can have a low melting point and are then termed ionic liquids (ILs). Thus, ILs are molten salts at the room temperature and consist of bulky organic cations paired with organic or inorganic anions. Imidazolium ionic liquids have many advantageous properties, such as no flammability, a wide electrochemical window, high thermal stability, wide liquid range, and very small vapor pressure [11]. They are also known to interact strongly with the basal plane of graphite and graphene. Polymeric imidazolium salts would therefore be interesting to explore as dispersing agents for graphene.

### Experimental

Graphene oxide was prepared by the modified Hummer's method [12,13]. The graphite flakes (PN 332461, 4 g; Sigma Aldrich, Sigma-Aldrich Sweden AB,) were first put in H_2_SO_4 _(98%, 12 mL) and kept at 80°C for 5 h. The resulting solution was cooled down to room temperature. Mild sonication was performed in a water bath for 2 h to further delaminate graphite into a few micron flakes. Sonication time and power are very critical as they define the size of the resulting graphene oxide sheets. Excessive sonication leads to extremely small flakes. Then, the solution was diluted with 0.5 L deionized (DI) water and left overnight. The solution was filtered by Nylon Millipore™ filters (Billerica, MA 01821). The resulting powder was mixed with KMnO_4 _and H_2_SO_4 _and put in a cooling bath under constant stirring for 1.5 h. The solution was diluted with DI water, and 20 mL H_2_O_2 _(30%) was added to it.

The supernatant was collected after 12 h and dispersed in dilute HCl in order to remove the metal ion residue and then recovered by centrifugation [12,13]. Clean GO was again dispersed in water to make a homogeneous dispersion and was centrifuged at 8,000 rpm for 40 min in order to remove the multilayer fragments. We added a polymeric imidazolium molten salt into the aqueous dispersion of GO at a concentrationof 1 mg mL^-1 ^and strongly shook the solution for a few minutes. The imidazolium salt used by us was polyquaternium 16 (PQ-16) sold under the trade name Luviquat Excellence by BASF **(Ludwigshafen, Germany)**, a copolymer with 95% molar of imidazolium chloride and 5% molar of vinylimidazole. Use of this polymeric salt for graphene dispersion is not found in literature. Then, the solution was reduced by hydrazine monohydrate at 90°C for 1 h to obtain a stable dispersion of graphene in aqueous solution.

## Results and discussion

**Figure 1:**
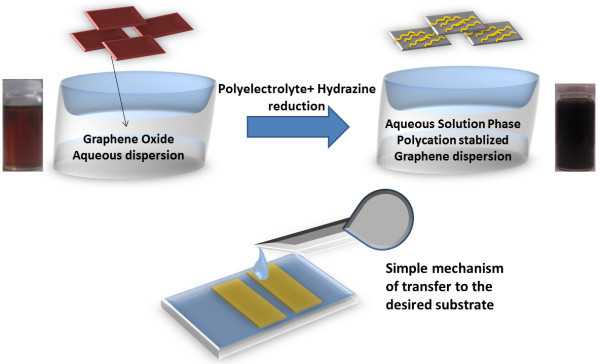
**Aqueous solutions of graphene oxide and graphene after hydrazine reduction**. In the presence of polyelectrolyte, schematic of the transfer mechanism.

**This aqueous PQ-graphene dispersion was found to be stable even after 2 months, whereas the reduced GO without the addition of PQ-16 formed agglomerates soon after reduction with hydrazine. Thus, PQ-16 is the main cause of a stable dispersion of graphene membranes in aqueous solution. The underlying mechanism has been affiliated with adsorption of some of the polycations on the surface of the graphene membranes by non-covalent π-π interactions between the imidazolium rings of the salt and graphene, soon after reduction with hydrazine monohydrate **[14]. The graphene was deposited onto Si/SiO_2 _(SiO_2 _thickness approximately 300 nm) substrates by dip-coating. Schematic of the whole process is shown in Figure 1.

The sample was rinsed with DI water and dried with nitrogen. The dried samples were further treated at 400°C for 2 h in Ar/H_2 _to further reduce the graphene oxide and also to sublimate the solution residue. The optical microscope images were taken in order to identify graphene [15]. Atomic force microscope measurements were carried out to confirm the presence of single- and few-layer graphene by measuring step height [7]. Graphene shows typical wrinkled structure which is intrinsic to graphene [16] over relatively large sheet sizes. Very large graphene membranes with sizes around 10 × 10 μm were identified. The size was found to be directly related with sonication power and time. Excessive sonication results in very small graphene sheets, whereas insufficient sonication results in incomplete exfoliation of graphite oxide.

**Figure 2:**
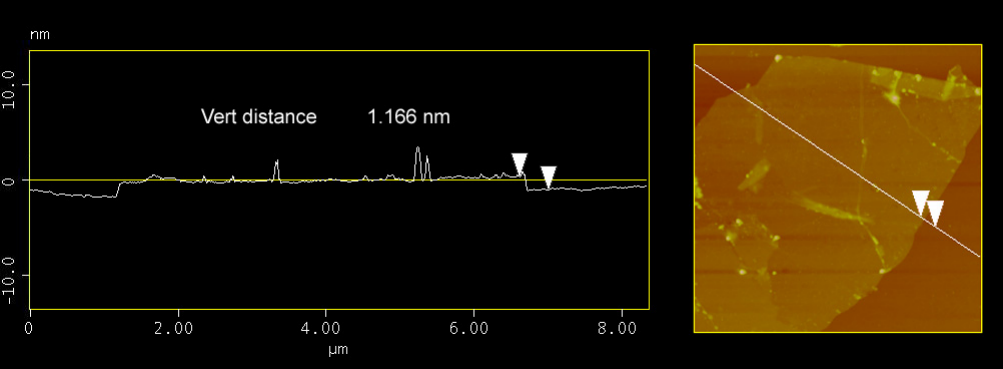
**Tapping mode AFM image of GO on SiO_2_/Si with step height profile**.

We measured the height profiles of the graphene membranes by atomic force microscopy (AFM) after drop casting them on a relatively flat SiO_2_/Si substrate. The average thickness of a GO sheet was approximately 1 nm (Figure 2), which was in agreement with the preceding research, confirming that the graphite oxide was completely exfoliated. We observed heights from slightly less than 1 nm to a few nanometers thick. We assigned the sheets with height approximately 1 nm, approximately 1.5 nm, approximately 2 nm, and up to 5 nm to be one-, two-, three-, and few-layered GO sheets, respectively. This was in agreement with the reported AFM results on few-layer graphene sheets [5,8,17], where the single-layer graphene is always approximately 1 nm, probably due to different attraction force between AFM tips and graphene as compared to SiO_2 _and imperfect interface between graphene and SiO_2_.

**Figure 3:**
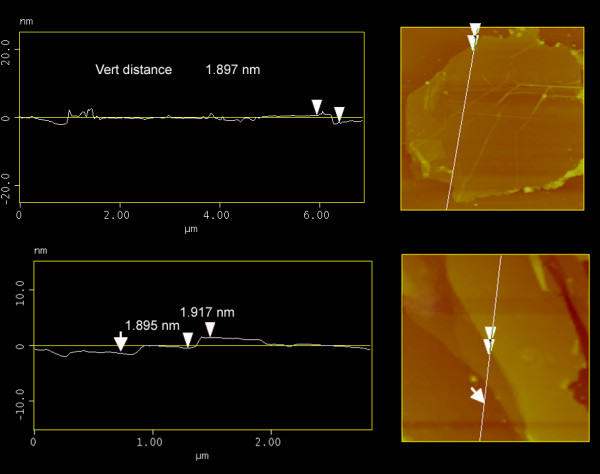
**AFM image of polyquaternium-stabilized graphene membrane with height profiles**.

AFM image of our chemically reduced GO sheet after addition of PQ-16, deposited on SiO_2_/Si substrate by drop casting, is shown in Figure 3. The graphite interlayer spacing is about 0.34 nm which should ideally correspond to the thickness of a monolayer graphene. Conversely, the thickness of single PQ-G was determined to be approximately 1.9 nm. If we assume that monolayered PQ-16 covered both sides of graphene sheet with offset face-to-face orientation via π-π interactions (mechanism of stabilization), the estimated distance between PQ and the graphene sheet is approximately 0.35 nm [18]. Accordingly, the average thickness of the graphene sheet in the PQ-G layer can be derived to be around 1.9 nm. This assumption is further supported by Figure 3b, which shows the step height for the region with bilayer graphene. The step height of the graphene-graphene interface was also observed to be approximately 1.9 nm in various measurements.

**Figure 4:**
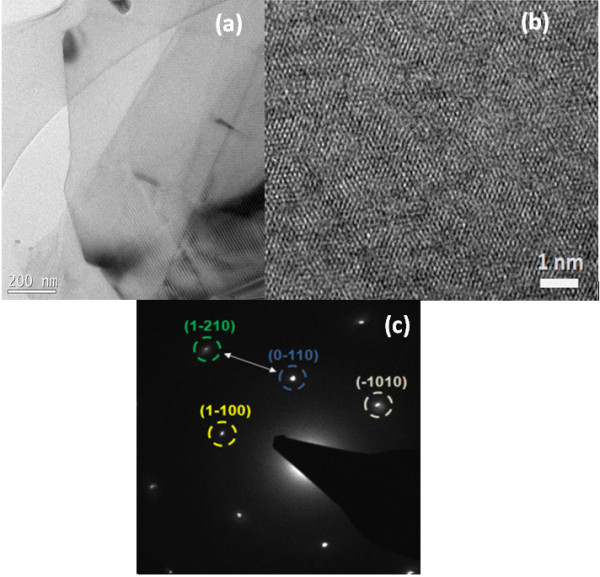
**Electron microscopy of graphene**. (**a**) Bright-field TEM images of monolayer graphene, (**b**) HRTEM image from the same location, and (**c**) electron diffraction pattern of the graphene sheet in (**a**) with diffraction spots labeled by Miller-Bravais indices.

Transmission electron microscopy (TEM) is also a very important tool for investigating the quality of exfoliated graphene. We dropped a small quantity of the dispersion on the holey carbon grid by pipette and dried the samples. Figure 4a shows bright-field TEM image, Figure 4b shows the high-resolution transmission electron microscope (HRTEM) image of the graphene surface, and Figure 4c depicts the electron diffraction pattern observed from the same area. The analysis of the diffraction intensity ratio was used to confirm the presence of monolayer graphene [19]. We use the Bravais-Miller (hkil) indices to label the peaks corresponding to the graphite reflections taken at normal incidence [19]. After analyzing a large number of TEM images, we were able to conclude that our dispersion contains a very good fraction of monolayer graphene.

**Figure 5:**
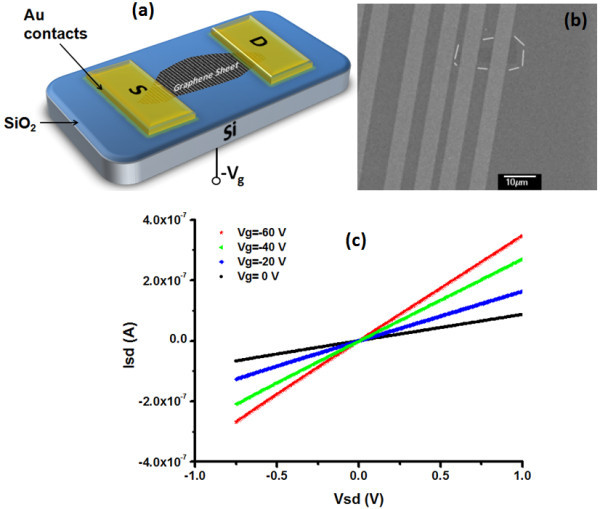
**Electronic devices based on reduced GO membrane**. (**a**) Schematic of a device with 30-nm-thick thermally evaporated Au contacts as the source (S) and drain (D) electrodes, (**b**) SEM image of the device, and (**c**) source-drain current (*I*_sd_) vs. source-drain voltage (*V*_sd_) as a function of gate voltage (*V*_g_) with p^++ ^silicon serving as a back gate.

We fabricated a bottom-gated graphene field-effect transistor (FET) by putting a monolayer of reduced GO membrane in between thermally evaporated gold electrodes. The channel length between source and drain electrodes was 5 μm. The schematic and the scanning electron microscope (SEM) image of the device are shown in Figure 5. Figure 5c shows the drain current (*I*_d_) vs. gate voltage (*V*_g_) curve of FET prepared with this reduced monolayer graphene membrane. The FET gate operation exhibits hole conduction behavior. Pure two-dimensional graphene has a zero bandgap that limits its effective application in electronic devices. We believe that this reduced GO from PQ dispersion has a kind of doping effect that makes it more favorable for applications due to its improved electronic properties. There were theoretical simulations [20,21], which were later confirmed experimentally [22] that the 100% hydrogenation of freestanding graphene results in a metal to insulator transition. Hydrogenation of graphene on a silicon dioxide (SiO_2_) substrate has also led to the energy gap opening [23]. Here, we can attribute the deficiency of ambipolar behavior to hole doping caused by residual oxygen functionalities resulting in a p-type behavior and a field-effect response [2,24]. Thus, chemical functionalization is a possible route to modify the electronic properties of graphene, which can be important for graphene-based nanoelectronics [25], although there is room for further optimization of the process for improving the properties, in order to make it ideal for industrial level applications.

## Conclusions

In summary, we report a method to produce and functionalize graphene membranes in the solution phase using polymeric imidazolium molten salts as a transferring medium. Graphene membranes were reduced from graphene oxide by hydrazine in the presence of a polyelectrolyte which was found to be a very stable dispersion for the graphene membranes in the aqueous solution. The reduced GO membranes were transferred to a SiO_2_/Si substrate by simple drop casting and were further reduced by annealing in H_2_/Ar. A simple device with gold contacts on both the sides was fabricated in order to observe the electronic properties. We conclude that chemical functionalization is a possible route to modify and improve the electronic properties of graphene.

## Competing interests

The authors declare that they have no competing interests.

## Authors' contributions

All authors contributed equally, read and approved the final manuscript.
